# 

**Published:** 1909-07-15

**Authors:** 


CONTENTS

Event and Comment - - - - - - - - - 335
A Plea for Consistency in the Use of Terms Applied to Local Remedies Classified According to Their Pharmacological Action, - - - *By E. T. Loeffler, B.S., D.D.S.* 336
Selection of a Location for Practice, - *By N. S. Hoff, D.D.S.* 344
New English Anesthetic Legislation, *By Edward C. Kirk, D.D.S., S. C. D.* 353
A Plea for Rationalism in Modern Methods of Practice *By C. N. Johnson, M.A., L.D.S., D.D.S.* 357
The Mouth in Its Relation to Stomach and Intestinal Diseases *By H. G. Langworthy, M.D.* 364
Indications for the Inlay - - *By J. F. Nyman, D.D.S.* 368
The Therapeutic Committee of the British Medical Association, *By C. N. LeBrocq, B.A., M.D.* 371
Indents - - - - - - - - - - - - 379

